# Fracture fermée du tibia associée à une luxation de chopart ipsilatérale: une entité clinique rare

**DOI:** 10.11604/pamj.2015.22.224.5144

**Published:** 2015-11-10

**Authors:** Atif Mechchat, Abdelmajid Elmrini

**Affiliations:** 1 Department of Orthopaedics and Trauma surgery B4, UH Hassan II-Fez, Morocco

**Keywords:** Fracture fermée, tibia, ipsilateral Chopart dislocation, close fracture, tibia, luxation de chopart ipsilatérale

## Image en medicine

Un patient de 22 ans se présente aux urgences pour une impotence fonctionnelle douloureuse et immédiate du pied gauche suite à un accident de la voie publique. L'examen clinique montre une vive douleur du tiers inférieur de la jambe, avec une cheville oedématiée sans ouverture cutanée. Les diagnostics à évoquer sont une fracture bimalléolaire ou une fracture du quart distale de jambe. Le bilan radiologique de la jambe face et profil ainsi que du trois-quarts déroulé du pied a objectivé une fracture transverse du tiers inférieur des deux os de la jambe gauche associée à une luxation homolatérale plantaire de l'articulation de chopart, soit une « cheville flottante ». Classiquement, une cheville flottante associe une fracture distale de jambe à une fracture du pied ipsilatérale créant ainsi une instabilité autour de la cheville laissant la mortaise intact. La luxation de l'articulation médiotarsienne (AMT) plantaire reste rare en raison des fortes structures ligamentaires en particulier le ligament plantaire, long et court, le ligament bifurqué et le ligament calcanéonaviculaire plantaire comme support de l'arche du pied. La luxation de l'articulation médiotarsienne plantaire est encore plus rare lorsqu'elle est associée à une fracture de jambe. Les chevilles flottantes sont des lésions rares, se présentant souvent dans le cadre de traumatismes ouverts, et dont le pronostic fonctionnel reste réservé. Le traitement se base sur l'immobilisation provisoire du membre traumatisé avant d'avoir recours à une ostéosynthèse adéquate des deux lésions.

**Figure 1:**
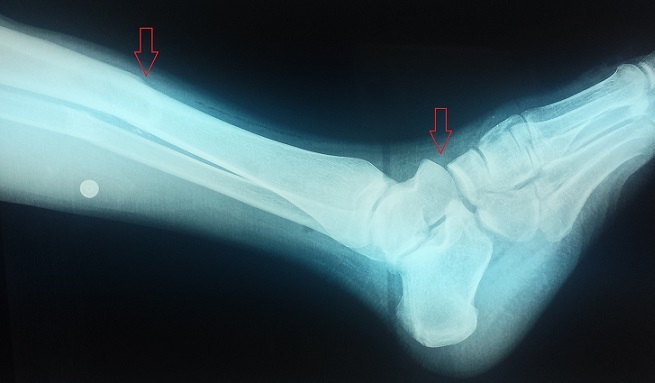
Fracture des deux os de la jambe médiodiaphysaire associée à une luxation plantaire de l'articulation de chopart

